# Contributions of pharmaceutical interventions to the multidisciplinary dysphagia team: A retrospective observational study

**DOI:** 10.1186/s40780-025-00474-x

**Published:** 2025-08-05

**Authors:** Akihito Ueda, Michiko Obara, Shinichi Watanabe

**Affiliations:** 1 Doctoral Program in Pharmaceutical Sciences, Graduate School of Pharmaceutical Sciences, Teikyo Heisei University, Tokyo, Japan; 2 Medical Corporation Toujinkai, Fujitate Hospital, 5-4-24 Omiya Asahi-Ku, Osaka-Shi, Osaka-Hu, 535-0002 Japan; 3 Faculty of Pharmaceutical Sciences, Teikyo Heisei University, Tokyo, Japan

**Keywords:** Dysphagia, Drug-induced dysphagia, Pharmaceutical intervention, Multidisciplinary team, Dopamine antagonist, Polypharmacy

## Abstract

**Background:**

The 2022 revision of Japanese healthcare reimbursement removed pharmacists from the mandatory dysphagia team, despite emerging evidence of medication-related swallowing complications. Our previous pharmacovigilance analysis identified dopamine-blocking drugs as primary contributors to the risk of aspiration pneumonia. This study aimed to validate these findings through a clinical examination of pharmaceutical interventions performed by a multidisciplinary dysphagia team.

**Methods:**

This retrospective observational study was conducted at a 97-bed community hospital in Osaka, Japan, from June 2023 to January 2024. All adult patients with suspected dysphagia who underwent a multidisciplinary team intervention were included in our analysis. Pharmaceutical intervention was requested when medication-related dysphagia or swallowing difficulties were suspected, with interventions classified into the following four categories: drug-induced dysphagia management, dosage form optimization, swallowing aid utilization, and medication burden reduction. Changes in the medication burden were analyzed using paired t-tests.

**Results:**

Among 59 patients with dysphagia (mean age, 81.1 ± 9.8 years; 33 males [55.9%], 26 females [44.1%]), 13 (22.0%) underwent pharmaceutical interventions. Drug-induced dysphagia management was the most common intervention (69.2%), targeting dopamine antagonists (sulpiride, risperidone, tiapride, and domperidone), benzodiazepines, and anticholinergics without dopamine-blocking effects. Suspected drug-induced dysphagia was the most common symptom among patients with dementia (38.9%). The intervention group showed a significant reduction in medication (mean, -3.2 medications; *P* < 0.001), whereas the non-intervention group showed no change. Among the non-intervention group, potential opportunities for the optimization of angiotensin-converting enzyme inhibitors were identified in antihypertensive therapy.

**Conclusions:**

Pharmaceutical interventions may offer clinically meaningful contributions when utilized for patients with dysphagia, supporting the relevance of pharmacovigilance regarding the risks of dopamine antagonists. The findings of this study suggest the importance of reinstating pharmaceutical expertise to multidisciplinary dysphagia teams, as pharmacists provide clinically significant medication optimization, including identifying additional optimization opportunities through systematic medication reviews among vulnerable populations.

## Background

Dysphagia affects many older adults and is associated with serious complications, including aspiration pneumonia, malnutrition, and mortality [1]. Recent systematic reviews report dysphagia prevalence of approximately 30% in community-dwelling elderly, 47% in nursing home residents, and 38% in hospitalized older adults [2]. Dysphagia is particularly challenging in Japan, given its unprecedented rapid aging trend. Traditionally, dysphagia management has involved a multidisciplinary team comprising physicians, nurses, speech-language pathologists, dietitians, and pharmacists [3]. However, the 2022 Japanese healthcare reimbursement revision removed pharmacists from the mandatory team composition for the “Dysphagia Function Recovery System Add-on,” following questioning about their contributions to the issue. 

However, pharmacists possess unique expertise for dysphagia management, including medication optimization and burden reduction. Evidence supports the need to identify medications that may contribute to dysphagia. Our recent pharmacovigilance analysis identified that among anticholinergic drugs, those with dopamine-blocking properties—antipsychotics in particular—are the primary contributors to the risk of aspiration pneumonia, rather than the anticholinergic effects themselves [4].

This study examined the pharmaceutical interventions of a multidisciplinary dysphagia team to validate their clinical relevance and demonstrate their value in dysphagia management.

## Methods

### Study design and setting

**Fig. 1:**
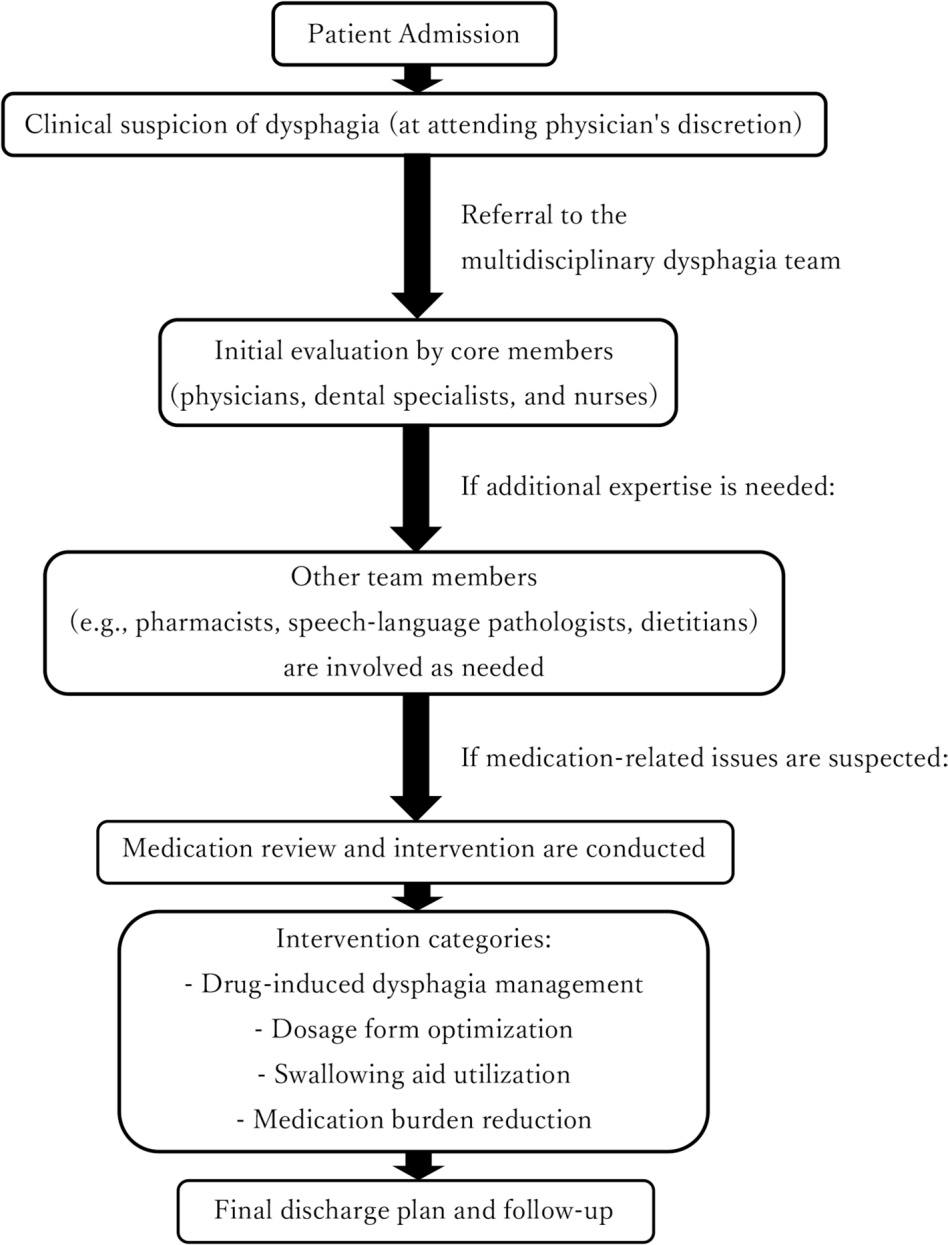
Workflow of the multidisciplinary dysphagia team intervention. Patients with suspected dysphagia are referred to the dysphagia team. Initial evaluation is conducted by core members (physician, dental specialist, and nurse). Other professionals (e.g., pharmacists, speech-language pathologists, dietitians) are involved as needed. When medication-related issues are suspected, a medication review and related intervention (as needed) are conducted. Interventions are categorized into four types

This retrospective observational study was conducted at Fujitate Hospital, a 97-bed community hospital in Osaka, Japan, between June 1, 2023, and January 31, 2024. The multidisciplinary dysphagia team involved a standard team including a Japanese Respiratory Society-certified physician with infection control expertise, a Japanese Society of Gerodontology-certified dental physician specializing in swallowing assessment, nurses, speech-language pathologists, dietitians, and pharmacists (Fig. 1), who intervened when medication-related issues were suspected.

### Participants

Our analysis included patients aged ≥ 18 years with suspected dysphagia who were referred to the multidisciplinary dysphagia team during the study period. Inclusion criteria were clinical suspicion of dysphagia necessitating team evaluation and complete medical records. Patients were classified into two groups: pharmaceutical intervention and non-intervention.

### Definitions

Medication-related dysphagia was defined as dysphagia suspected to be caused or exacerbated by medications that can impair swallowing function through dopaminergic blockade, central nervous system depression, or anticholinergic effects [5].

### Data collection and analysis

Data extracted from the patients’ electronic medical records included demographic information, primary diagnosis, dysphagia etiology, admission and discharge medication profiles, pharmaceutical interventions, and clinical outcomes. Pharmaceutical interventions were classified into the following four categories: management of suspected drug-induced dysphagia (including drug discontinuation), dosage form optimization, swallowing aid utilization, and medication burden reduction for polypharmacy optimization. The primary outcome was the proportion of patients receiving pharmaceutical interventions. Secondary outcomes included intervention types, medication burden changes, and patient characteristics. Additional data collection included discontinued medications (classified by drug category), drug-induced dysphagia (stratified by underlying etiology), and identification of potential pharmaceutical intervention opportunities among non-intervention cases through systematic medication review.

### Statistical analysis

Changes in medication burden between admission and discharge were analyzed using paired t-tests, with statistical significance set at *P* < 0.05. Statistical analyses were performed using JMP Pro software (version 17.0; SAS Institute Inc., Cary, NC, USA).

## Results

### Patient characteristics

Overall, 59 patients with dysphagia were included. Their demographic and clinical characteristics are presented in Table 1.

**Table 1:** Patient Demographics and Clinical Characteristics (*n* = 59)

Characteristic	Result
Age (mean ± standard deviation)	81.1 ± 9.8 years
Male/Female	33 (55.9%) vs. 26 (44.1%)
**Primary Diagnosis**	
Aspiration pneumonia	27 (45.8%)
Coronavirus disease 2019 (COVID-19) infection	7 (11.9%)
Chronic heart failure	2 (3.4%)
Urinary tract infection	2 (3.4%)
Dementia	2 (3.4%)
Other	19 (32.2%)
**Dysphagia Etiology**	
Cerebrovascular disease	18 (30.5%)
Dementia	18 (30.5%)
Neurological/neuromuscular disease	7 (11.9%)
Psychiatric disorders	4 (6.8%)
Respiratory disease	2 (3.4%)
Other	10 (16.9%)
**Discharge Outcomes**	
Facility placement	24 (40.7%)
Transfer to another hospital	14 (23.7%)
Home discharge	12 (20.3%)
Death	9 (15.3%)

^*^Values are presented as mean ± *standard deviation or number (%), as appropriate*

### Pharmaceutical interventions

Thirteen (22.0%) patients underwent pharmaceutical interventions. Their characteristics are stratified by intervention status in Table 2.

**Table 2:** Patient Characteristics by Pharmaceutical Intervention Status

Characteristic	Pharmaceutical Intervention (*n* = 13)	No Pharmaceutical Intervention (*n* = 46)
Age (mean ± SD)	83.1 ± 8.7 years	80.5 ± 10.2 years
Male/Female	6 (46.2%) vs 7 (53.8%)	27 (58.7%) vs 19 (41.3%)
Primary Diagnosis	Aspiration pneumonia: 7 COVID-19 infection: 1 Dementia: 1 Other: 4	Aspiration pneumonia: 20 COVID-19 infection: 6 Chronic heart failure: 2 Urinary tract infection: 2 Dementia: 1 Other: 13
Dysphagia Etiology	Cerebrovascular disease: 3 Dementia: 7 Psychiatric disorders: 1 Respiratory disease: 1 Other: 1	Cerebrovascular disease: 15 Dementia: 11 Neurological/neuromuscular disease: 7 Psychiatric disorders: 3 Respiratory disease: 1 Other: 9
Discharge Outcomes	Facility placement: 7 Transfer to another hospital: 3 Home discharge: 3	Facility placement: 17 Transfer to another hospital: 11 Home discharge: 9

*Values are presented as mean* ± *standard deviation or number (%), as appropriate*

Drug-induced dysphagia management (69.2%) and dosage form optimization (15.4%) were most common (Table 3).

**Table 3:** Pharmaceutical Intervention Categories (*n* = 13)

Category	Cases (%)
Drug-induced dysphagia management	9 (69.2%)
Dosage form optimization	2 (15.4%)
Swallowing aid utilization	1 (7.7%)
Medication burden reduction	1 (7.7%)

*Values are presented as number (%)*

The medications targeted for discontinuation included dopamine antagonists (sulpiride, risperidone, tiapride, and domperidone), benzodiazepines (flunitrazepam, etizolam, alprazolam, and quazepam), anticholinergics without dopamine-blocking effects (solifenacin), and antitussives (dextromethorphan).

Suspected drug-induced dysphagia was more frequent among patients with dementia (7/18 cases, 38.9%) than those with psychiatric (1/4 cases, 25.0%) or cerebrovascular (1/18 cases, 5.6%) diseases (Table 4).

**Table 4:** Drug-Induced Dysphagia by Etiology

Dysphagia Etiology	Drug-Induced Cases	Total Cases
Dementia	7	18
Psychiatric disorders	1	4
Cerebrovascular disease	1	18
Neurological/neuromuscular disease	0	7
Respiratory disease	0	2
Other	0	10
**Total**	**9**	**59**

*Values are presented as number (%)*

### Impact on medication burden

Among the 50 patients who survived to discharge, the intervention group showed a significant medication reduction from admission to discharge (mean reduction, −3.2 medications; *P* < 0.001), primarily because of discontinuations of drugs in the drug-induced dysphagia management category, whereas the non-intervention group showed no reduction (Table 5).

**Table 5:** Medication Burden Changes (Admission → Discharge)

Group	Cases	Admission	Discharge	Change	*P*-value
Pharmaceutical intervention	13	6.3 ± 2.8	3.1 ± 3.0	−3.2 ± 2.5	< 0.001
No pharmaceutical intervention	37	6.8 ± 3.9	6.9 ± 3.6	+ 0.1 ± 2.3	Not significant
**Overall**	**50**	**6.7** ± 3.6	**5.9** ± 3.9	**−0.8** ± 2.8	**–**

*Values are presented as mean* ± *standard deviation*

*P-values were calculated using paired t-tests*

*“Not significant” indicates P* ≥ *0.05*

### Potential intervention opportunities in non-intervention cases

Among the 46 patients who did not undergo pharmaceutical interventions, potential optimization opportunities were revealed through antihypertensive therapy investigation. Of the 22 patients receiving antihypertensive medications, only 3 were prescribed angiotensin-converting enzyme (ACE) inhibitors or angiotensin II receptor blockers (ARB) (ACE inhibitor monotherapy, *n* = 2; ARB monotherapy, *n* = 1), while 19 received calcium channel blocker-based regimens. Among the 6 patients in the intervention group who were undergoing antihypertensive therapy, only 1 was switched to an ACE inhibitor.

## Discussion

While previous research has discussed the importance of multidisciplinary approaches for dysphagia management, detailed reports on pharmacists’ specific contributions remain scarce. This study addresses this gap by providing comprehensive data on pharmaceutical interventions in multidisciplinary dysphagia care. Our findings demonstrate that pharmaceutical interventions provided meaningful contributions to 22% of the patients with dysphagia cared for by a multidisciplinary team, achieving substantial medication optimization while maintaining therapeutic goals. Although direct comparisons with previous studies were limited owing to scarce literature on pharmaceutical intervention rates in dysphagia teams, this finding highlights the potential role of pharmaceutical expertise. The significant reduction in medication (mean, −3.2 medications) validates the importance of pharmaceutical expertise in multidisciplinary dysphagia management.

### Drug-induced dysphagia in vulnerable populations

The high frequency of suspected drug-induced dysphagia among patients with dementia (38.9%) highlights this population’s vulnerability to medication-related swallowing complications. Our findings suggest that psychotropic medications are often unnecessarily continued in patients with dementia, even after symptom resolution [6, 7]. All patients in our study were evaluated by psychiatrists before attempting medication reduction. Through appropriate pharmaceutical interventions and psychiatric supervision, many of these medications could be discontinued, leading to improved swallowing function. This underscores the importance of regular medication reviews and deprescribing approaches in this population.

In contrast, patients with psychiatric disorders showed limited medication reduction due to the risk of underlying psychiatric condition exacerbation. This reflects the challenge of balancing therapeutic benefits against dysphagia risks, as drug-induced dysphagia persisted despite intervention, highlighting the need for alternative strategies when deprescription is unfeasible.

### Pharmaceutical intervention strategies

This study illustrates various approaches for medication optimization among patients with dysphagia. Dosage form optimization includes conversion to orally disintegrating tablets (ODT) and transdermal formulations [8]; however, ODT may accumulate in the pharynx of patients with severe dysphagia, emphasizing the need for individualized assessment [9], e.g., through video endoscopy.

### Clinical relevance to previous pharmacovigilance analysis findings

Our findings validate and extend our previous pharmacovigilance research [4], which suggested that pharmacological effects other than anticholinergic action, particularly dopamine antagonism and central nervous system depression, may play a more important role in the risk of aspiration pneumonia. This study provides clinical confirmation of this association, with dopamine antagonists and benzodiazepines being the most frequently discontinued medications among patients with suspected drug-induced dysphagia. This sequential research approach, from database analysis to clinical validation, strengthened the evidence of specific medication-related risk factors in dysphagia management. The discontinuation of dopamine antagonists in our clinical practice directly aligns with the JADER analysis findings [4]. The high intervention rate among patients with dementia further corroborates the vulnerability of this population, seen in our pharmacoepidemiologic analysis. This convergence between pharmacovigilance signals and real-world clinical outcomes demonstrates the value of database analysis in guiding therapeutic decision-making and validates the mechanistic hypothesis that dopaminergic blockade, rather than anticholinergic effects alone, represents the primary pathophysiological mechanism underlying drug-induced dysphagia.

### Potential for additional interventions

Reviewing the medication profiles revealed opportunities for pharmaceutical interventions beyond those identified based on clinical suspicion. Among the 22 patients undergoing antihypertensive therapy, only 2 were prescribed ACE inhibitors, which enhance substance P levels and improve cough reflex sensitivity, reducing aspiration pneumonia risk in elderly patients [10, 11]. Recent comprehensive reviews have further confirmed these preventive effects and expanded therapeutic strategies for aspiration pneumonia in older adults [12].

Medication reduction substantially decreased polypharmacy burden, potentially reducing the risk of adverse drug reactions [13] and facilitating more effective swallowing rehabilitation. This analysis underscores the importance of systematic pharmaceutical reviews in dysphagia management, extending beyond reactive interventions to proactive medication optimization.

### Study limitations

This study had some limitations. First, as a small single-center retrospective descriptive study without a control group, generalizability may be restricted, and causal relationships between interventions and outcomes cannot be established. Second, standardized criteria for the assessment of drug-induced dysphagia are lacking, potentially introducing subjective elements into the diagnosis. Finally, pharmaceutical interventions were requested based on clinical necessity, potentially introducing selection bias.

Future research should focus on developing standardized assessment tools for drug-induced dysphagia, conducting multicenter studies with larger populations, and implementing prospective trials to evaluate systematic pharmaceutical interventions to prevent aspiration pneumonia.

## Conclusions

Pharmaceutical interventions may offer clinically meaningful contributions to patients with dysphagia cared for by a multidisciplinary team, achieving substantial medication optimization while maintaining therapeutic efficacy. These findings validate pharmacovigilance evidence regarding dopamine antagonist risks and indicate the importance of pharmaceutical expertise in multidisciplinary dysphagia management. The high frequency of suspected drug-induced dysphagia among patients with dementia emphasizes the need for systematic medication reviews in this vulnerable population. These findings support advocacy for reintegrating pharmaceutical expertise into Japanese dysphagia team reimbursement through evidence-based demonstration of clinical value and cost-effectiveness. 

## Declaration of generative AI and AI-assisted technologies in the writing process

Generative AI (Claude Sonnet 4, Anthropic) was used to enhance language clarity and structure during the preparation of the manuscript. All of the authors reviewed the content thoroughly and take full responsibility for its accuracy and integrity. 

## Data Availability

No datasets were generated or analysed during the current study.

## Abbreviations

ACE: Angiotensin-converting enzyme
ARB: Angiotensin II receptor blockers
COVID-19: Coronavirus disease 2019
ODT: Orally disintegrating tablets
